# Salivary IL-6 mRNA is a Robust Biomarker in Oral Squamous Cell Carcinoma

**DOI:** 10.3390/jcm8111958

**Published:** 2019-11-13

**Authors:** Ildikó Judit Márton, József Horváth, Péter Lábiscsák, Bernadett Márkus, Balázs Dezső, Adrienn Szabó, Ildikó Tar, József Piffkó, Petra Jakus, József Barabás, Péter Barabás, Lajos Olasz, Zsanett Kövér, József Tőzsér, János Sándor, Éva Csősz, Beáta Scholtz, Csongor Kiss

**Affiliations:** 1 Department of Operative Dentistry and Endodontics, Faculty of Dentistry, University of Debrecen, 4032 Debrecen, Hungary; 2 Department of Biochemistry and Molecular Biology, Faculty of Medicine, University of Debrecen, 4032 Debrecen, Hungary; horvathjozsef21@gmail.com (J.H.); labiscsak.peter@med.unideb.hu (P.L.); jakob.bernadett@med.unideb.hu (B.M.); tozser@med.unideb.hu (J.T.); cseva@med.unideb.hu (É.C.); scholtz@med.unideb.hu (B.S.); 3 Department of Oral Pathology and Microbiology, Faculty of Dentistry and Institute of Pathology, University of Debrecen, 4032 Debrecen, Hungary; dezsob51@gmail.com; 4 Department of Maxillofacial Surgery, University of Debrecen, 4032 Debrecen, Hungary; szabo.adrienn@dental.unideb.hu; 5 Department of Oral Medicine, Faculty of Dentistry, University of Debrecen, 4032 Debrecen, Hungary; tar.ildiko@dental.unideb.hu; 6 Department of Maxillofacial Surgery, University of Szeged, 6720 Szeged, Hungary; piffkojozsef@gmail.com (J.P.); jakus.petra.87@gmail.com (P.J.); 7 Department of Maxillofacial Surgery, Semmelweis University, 1085 Budapest, Hungary; barabas.jozsef@dent.semmelweis-univ.hu (J.B.); dr.barabas.peter@gmail.com (P.B.); 8 Department of Oral and Maxillofacial Surgery, University of Pécs, 7621 Pécs, Hungary; olasz.lajos@pte.hu (L.O.); kover.zsanett@pte.hu (Z.K.); 9 Department of Preventive Medicine, University of Debrecen, 4028 Debrecen, Hungary; sandor.janos@sph.unideb.hu; 10 Department of Pediatric Hematology-Oncology, Faculty of Medicine, University of Debrecen, 4032 Debrecen, Hungary; kisscs@med.unideb.hu

**Keywords:** salivary biomarkers, oral neoplasia, periodontal disease/periodontitis, smoking, ethanol consumption, real-time quantitative PCR, enzyme-linked immune-sorbent assay, immunohistochemistry

## Abstract

Salivary IL-6 mRNA was previously identified as a promising biomarker of oral squamous cell carcinoma (OSCC). We performed a multi-center investigation covering all geographic areas of Hungary. Saliva from 95 patients with OSCC and 80 controls, all Caucasian, were collected together with demographic and clinicopathological data. Salivary IL-6 mRNA was quantified by real-time quantitative PCR. Salivary IL-6 protein concentration was measured by enzyme-linked immune-sorbent assay. IL-6 protein expression in tumor samples was investigated by immunohistochemistry. Normalized salivary IL-6 mRNA expression values were significantly higher (*p* < 0.001) in patients with OSCC (mean ± SE: 3.301 ± 0.885) vs. controls (mean ± SE: 0.037 ± 0.012). Differences remained significant regardless of tumor stage and grade. AUC of the ROC curve was 0.9379 (*p* < 0.001; 95% confidence interval: 0.8973–0.9795; sensitivity: 0.945; specificity: 0.819). Salivary IL-6 protein levels were significantly higher (*p* < 0.001) in patients (mean ± SE: 70.98 ± 14.06 pg/mL), than in controls (mean ± SE: 12.45 ± 3.29). Specificity and sensitivity of IL-6 protein were less favorable than that of IL-6 mRNA. Salivary IL-6 mRNA expression was significantly associated with age and dental status. IL-6 manifestation was detected in tumor cells and tumor-infiltrating leukocytes, suggesting the presence of a paracrine loop of stimulation. Salivary IL-6 mRNA is one of the best performing and clinically relevant biomarkers of OSCC.

## 1. Introduction

Hungarian males and females exhibit the highest age-standardized rates both for the incidence and the mortality of oral cavity and pharyngeal cancers in Europe without substantial improvements in the last decades [1]. Frequently, oral squamous cell carcinoma (OSCC) is being diagnosed in advanced stages, i.e., stage III and IV, with long-term survival rates around 50% despite considerable progress in surgical methods, radio-, chemo-, and immunotherapy. In contrast, patients with early, i.e., stage I and II lesions, may experience recovery rates up to 80%. Unfortunately, with the exception of the gold standard procedure, tissue biopsy and histopathological analysis, there are no evidence-based, reliable, non-invasive methods for large-scale screening and early detection of OSCC [2,3].

The aim of the present investigation was to study the potential of salivary IL-6 mRNA and IL-6 protein as OSCC-related biomarkers. Saliva was chosen as a complex, informative body-fluid containing biomolecules that originate from multiple sources and may differentiate between healthy subjects and patients with OSCC. Application of advanced molecular methods resulted in the discovery of several candidate salivary proteins, metabolites, mRNAs, miRNAs, and circRNAs associated with OSCC. However, there has been a high degree of variation between results reported by different investigators with respect to the value of these biomolecules as biomarkers detected in patients of different geographic and ethnic backgrounds [4,5,6,7,8,9,10]. In addition, salivary levels of inflammatory cytokines, considered as one of the best performing molecular groups of OSCC biomarkers, may be confounded by the presence of oral inflammatory lesions [11,12,13].

Previously, we applied both targeted and high-throughput molecular methods to identify potentially useful mRNA and protein biomarkers in a small-scale single-institution pilot cohort of patients with OSCC and controls [12,13]. These pilot transcriptomic and proteomic investigations suggested that salivary IL-6 mRNA and protein may prove the best performing biomarkers of OSCC in the Hungarian population. Therefore, we investigated salivary IL-6 mRNA and IL-6 protein in a sample of patients with OSCC covering each geographic area of Hungary. To support the relevance of salivary IL-6 mRNA expression, we were looking for differences in salivary IL-6 protein concentrations of patients and controls and by detecting the expression of IL-6 protein in tumor cells and in tumor-infiltrating leukocytes (TIL). An additional objective was to analyze associations of salivary IL-6 mRNA and protein expression with age, sex, gingival inflammation status, smoking, and ethanol consumption habits in patients with OSCC.

## 2. Materials and Methods

### 2.1. Patients and Control Subjects

Between 2 May 2013 and 31 December 2015, 95 adult (>18 years) patients with OSCC, presenting a suitable saliva sample, were enrolled in a multi-centric investigation from four Hungarian sites: Faculty of Dentistry, University of Debrecen, Debrecen (“Debrecen Center”): 26 patients; Faculty of Dentistry, Scientific University of Szeged, Szeged (“Szeged Center”): 24 patients; Faculty of Dentistry, Scientific University of Pécs, Pécs (“Pécs Center”): 25 patients; and Faculty of Dentistry, Semmelweis University, Budapest (“Budapest Center”): 20 patients. Eighty age-matched adult controls, admitted for dental check-ups, were recruited from the same sites. Subjects with previous and present cancer, except for the present OSCC (patients), coexisting diabetes, autoimmune disorders, contagious diseases, and pregnancy were excluded. 

The investigation was approved by the Institutional Review Board of the University of Debrecen (No. 3244–8/2011, No. 3722–2012) and by the Scientific and Research Ethics Committee, Medical Research Council, Hungary [(693/PI/12.) 45038-1/2012/EKU]. The Code of Ethics of the World Medical Association and the ethical standards of the 2000 Revision of the Helsinki Declaration were respected. Signed informed consent was obtained from all participating subjects. 

Examination of the oral cavity and the head and neck region was performed by a licensed dental and/or maxillofacial surgeon according to standard methods and criteria of the World Health Organization [14]. Tooth decay was characterized using the DMFT index by calculating the number of decayed (D), missing (M), and filled (F) teeth (T). Periodontal health was characterized by the gingival index (GI) [15]. Suspicious lesions were removed or biopsied, and OSCC was verified by histology investigating the hematoxylin- and eosin-stained slides. The histological differentiation grade of OSCC was defined according to the classification of the World Health Organization (WHO) [16]. The staging was performed according to the 8th edition of the TNM classification of the International Union Against Cancer [17]. Demographic and clinicopathological data, including age, sex, stage, and grade of OSCC, DMFT, and GI indices, were recorded. The stage and grade of OSCC lesions were not available from study subjects of the Pécs Center. Smoking and ethanol consumption habits were recorded from consenting subjects as precisely as possible. Based on the information obtained, we formed two groups in each category. Regarding smoking habits, we distinguished regular smokers and non-smokers/occasional smokers (<10 cigarettes/day). Regarding ethanol consumption habits, we formed two categories: people who consumed ethanol-containing beverages at least once a week vs. those who drank less than once a week.

### 2.2. Saliva Collection and Processing

Unstimulated saliva samples were collected between 9:00 and 11:00 a.m. Patients and controls refrained from eating, drinking, smoking, gum chewing, and performing oral hygiene measures for at least 60 min before sampling. A minimum of 5.0 mL saliva was collected from each participant. Samples were kept on ice throughout collection and processing. Samples were pushed five-times through a 19 g needle then filtered (Millex 25 mm Durapore PVDF 5 µm Sterile, Merck KGaA, Darmstadt, Germany). Three aliquots of 200 µL filtered saliva were mixed with 1 mL PAXgene^®^ reagent in cryotubes, for immediate stabilization of salivary RNA (PAXgene^®^ Blood RNA Tubes, BD Biosciences, Cat.No. 762165, BD Franklin Lakes, NJ, USA). Aliquots of 0.5 mL were pipetted from the remaining saliva filtrate into cryotubes, for ELISA (Cryopure tube, SARSTEDT, Cat.No. 72.380, Nümbrecht, Germany). The PAXgene^®^-stabilized and proteomic saliva samples were frozen within 60 min from collection and stored at −70 °C until processing. 

### 2.3. Reverse Transcription and Real-Time Quantitative PCR (qPCR) 

Salivary RNA was isolated with the Direct-zol™ RNA MiniPrep Plus Kit (Zymo research, Cat.No. R2072, Irvine, CA, USA) according to the manufacturer’s instructions. RNA sample quality and concentration were characterized by Nanodrop spectrophotometry and by the Agilent 2100 Bioanalyzer system. Expression of IL-6 mRNA and two normalizing genes, GAPDH and ACTB, were quantified using qPCR with TaqMan^®^ assays on a QuantStudio™ 12K Flex Real Time PCR System (Applied Biosystem^®^, Thermo Fisher Scientific, Waltham, MA, USA). Raw data analysis was performed using the ExpressionSuite Software v1.0.3 (Applied Biosystem^®^). Samples with a high standard deviation of replicates, or with only one replicate giving a signal in the qPCR assay were categorized as borderline, non-quantifiable-positive ones. Ct values of the latter samples were set uniformly to 39 for the calculations and statistical analysis. 

For qPCR efficiency correction, raw Ct values of cDNA serial dilutions (from HeLa total RNA) were used to determine the slope of the assays, using the linear regression function of GraphPad Prism software (GraphPad Software, Inc., CA, USA). PCR efficiency values were calculated, Ct values were corrected, and data were normalized. qPCR efficiencies were as follows: IL-6 = 0.904, GAPDH = 0.814, ACTB = 1.03. Cutoff Ct values (limit of quantitation; LOQ) for each TaqMan^®^ assay were also determined in these experiments. Detailed descriptions of RNA methods were provided as Supplementary data.

### 2.4. Enzyme-Linked Immune-Sorbent Assay (ELISA)

IL-6 protein concentration in saliva samples of patients and controls was determined in duplicates by the sandwich ELISA kit (Human ELISA Kit EK0410, Boster Biological Technology Co., Pleasanton, CA, USA) according to the manufacturer’s instructions. Optical density was measured at 450 nm, and concentrations were calculated based on the recorded 7-point calibration curve. LOQ for IL-t6 protein concentration was 4.96 pg/mL.

### 2.5. Immunohistochemistry (IHC) 

Sections obtained from 41 preexisting formalin-fixed and paraffin-embedded tissue blocks of patients with proven OSCC from the Debrecen Center were microscopically reviewed by an independent pathologist (BD). Since IHC labeling of preexisting tissue blocks did not interfere with salivary IL-6 mRNA and protein measurements, 15 samples from the previous pilot cohort were co-investigated with the 26 samples collected in the course of the present investigation to increase sample size [12,13]. Serial sections were used for the detection of IL-6 manifestation in tumor cells and TILs by IHC, as described in detail [18]. Briefly, mouse monoclonal antibodies (MoAb, clone 8H12; Invitrogen, Rockford, IL, USA) to IL-6 (8H12; Invitrogen; Thermo Fisher Scientific, Rockford, IL, USA), CD3, CD4, CD20, and CD163 (all from DAKO, Glostrup, Denmark) were used according to manufacturers’ instructions. Peroxidase-conjugated anti-mouse secondary immunoglobulin was used with a peroxidase-based detection kit (DAKO, Glostrup, Denmark) and VIP or DAB chromogenic substrates (Vector Labs, Peterborough, UK) for visualization. Stained sections were digitalized using a Panoramic MIDI digital slide scanner (3D-Histech-Zeiss, Budapest, Hungary) equipped with a Hitachi (HV-F22CL) 3CCD camera. Image analysis was performed by the HistoQuant application of Panoramic viewer software 1.15.2 (3D-Histech, Budapest, Hungary) as described [19].

### 2.6. Statistical Analysis

Distribution of demographic characteristics and clinical parameters of patients and controls were described by mean values and standard deviations (SD) for continuous variables. One-way ANOVA for continuous and chi-square test for non-continuous variables were used to check for uneven distribution of patients’ parameters between controls and cases. Statistical computation was carried out by IBM SPSS Statistics version 20 (IBM SPSS Statistics for Windows, Version 20.0, Armonk, NY, USA).

In describing the expression of IL-6 mRNA and IL-6 protein, mean values and standard errors (SE) were computed. Differences between patients and controls were assessed with GraphPad Prism, using the Mann-Whitney U-test on the efficiency-corrected, normalized IL-6 mRNA expression values, and IL-6 protein concentration values. 

Receiver Operating Characteristic (ROC) curve analysis was carried out on the same data with GraphPad Prism to evaluate the discrimination properties of IL-6 mRNA and IL-6 protein, and the area under the curve (AUC) was determined.

Multivariate linear regression models were applied to investigate the influence of patients’ age, sex, DMFT score, GI score, smoking, and ethanol consumption on IL-6 mRNA and IL-6 protein concentrations. Before modeling, IL-6 mRNA and IL-6 protein concentrations were ln-transformed because of these parameters showed right-tailed distribution. The normal distribution of ln transformed values was checked by the Kolmogorov-Smirnov test. Results were described by linear regression coefficients with the corresponding p-values. Statistical computation was carried out by IBM SPSS Statistics version 20.

## 3. Results

### 3.1. Characteristics of Study Participants

Demographic and clinicopathological characterization of 95 patients with newly diagnosed and histologically-verified OSCC and 80 age-matched controls were summarized in Table 1. There were more males (*N* = 60) than females (*N* = 35) among patients with OSCC, resulting in a significant difference in sex distribution between the patient and control groups. The patient group was characterized by significantly higher DMFT scores than the group of controls. Smoking and ethanol consumption were significantly more frequent among patients than controls. Both IL-6 mRNA and IL-6 protein determination were successfully performed in 53 patient samples. Twenty-one and 21 samples, each, from different patients were suitable for either IL-6 mRNA or IL-6 protein measurements. In the case of the 80 controls, the number of successful salivary analyses were as follows, IL-6 mRNA and IL-6 protein: 55, IL-6 mRNA only: 9, and IL-6 protein: only 16 (Table S1: Demographic, clinicopathological characterization, and IL-6 mRNA and IL-6 protein levels of individual patients with OSCC and controls).

### 3.2. Salivary IL-6 mRNA and IL-6 Protein Expression

**Figure 1:**
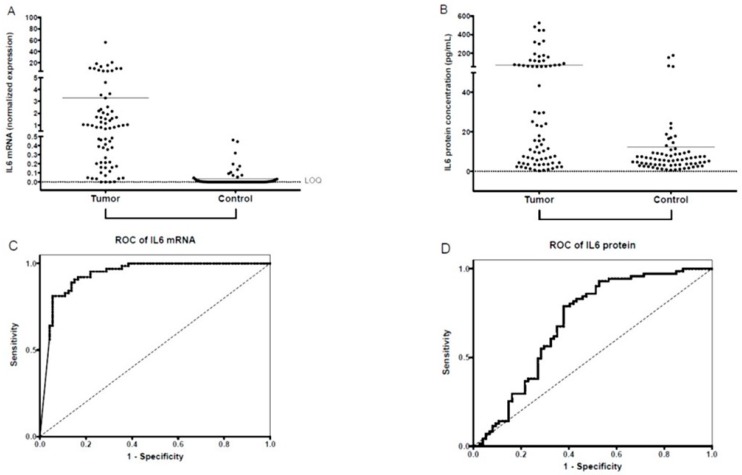
Salivary IL-6 mRNA and IL-6 protein expression. Expression of normalized salivary IL-6 mRNA (**A**) and salivary IL-6 protein (**B**) in patients with OSCC and controls. The horizontal lines indicate the mean. The p values are derived from the Mann-Whitney U test. Differences between Tumor and Control groups proved significant at *p* < 0.001 for both salivary IL-6 mRNA normalized values and for salivary IL-6 protein concentrations (*p* < 0.05 was considered significant). Dotted lines indicate the limit of quantification (LOQ). Receiver operating characteristic (ROC) curves for diagnostic ability of IL-6 mRNA (**C**) and protein (**D**) levels in saliva for OSCC. AUC for IL-6 mRNA = 0.9379 (*p* < 0.001; 95% confidence interval: 0.8973–0.9795; sensitivity: 0.945; specificity: 0.819); AUC for IL-6 protein = 0.6981 (*p* < 0.0001; 95% confidence interval: 0.6105–0.7858; sensitivity: 0.622; specificity: 0.789).

Saliva samples of 74/95 patients and 64/80 controls were suitable for qPCR. The majority of saliva samples from patients were positive for IL-6 mRNA. Initially, there were 18 samples with no quantifiable results. Reverse transcription and qPCR of these cases were repeated, resulting in 71 quantifiable results of 74 IL-6 mRNA samples from patients with OSCC. In contrast, 46 of 64 control samples were non-quantifiable (36 negative, and 10 borderline positive samples) for IL-6 mRNA expression, even in the repeated measurements. Normalized salivary IL-6 mRNA expression values were significantly higher in the group of patients with OSCC than in controls. The difference remained significant when subgroups of patients with different stages and grades were compared with controls (Table 2, Figure 1A). There were no significant differences in IL-6 mRNA expression between subgroups of patients with different stages and grades (data not shown). To test the sensitivity and specificity of salivary IL-6 mRNA as a diagnostic biomarker for OSCC, an ROC curve analysis was performed, and the AUC was calculated. The AUC for IL-6 mRNA was 0.9379 (*p* < 0.001; 95% confidence interval: 0.8973–0.9795; sensitivity: 0.945; specificity: 0.819) (Figure 1C).

IL-6 protein concentration was measured in 74 suitable salivary samples of 95 patients and in 71/80 controls. Patients with OSCC, both the total patient group and subgroups of patients according to stages and grades, had statistically significantly higher IL-6 concentrations than controls (Table 2, Figure 1B). There were no significant differences in IL-6 protein concentrations of subgroups of patients with different stages and grades. There was a partial overlap between the distribution of the salivary IL-6 protein concentration of the patient and the control groups (Figure 1B). Using ROC analysis, salivary IL-6 protein concentration successfully identified patients with OSCC. The AUC for IL-6 protein was 0.6981 (*p* < 0.0001; 95% confidence interval: 0.6105–0.7858; sensitivity: 0.622; specificity: 0.789) (Figure 1D).

According to the multivariate linear regression analysis, the age and DMFT score had significant associations with ln transformed IL-6 mRNA expression levels. Sex, GI scores, alcohol consumption, and smoking exhibited no statistically significant association either with salivary IL-6 RNA or IL-6 protein expression (Table 3).

### 3.3. IL-6 Protein Expression in OSCC Tumor Tissue

**Figure 2:**
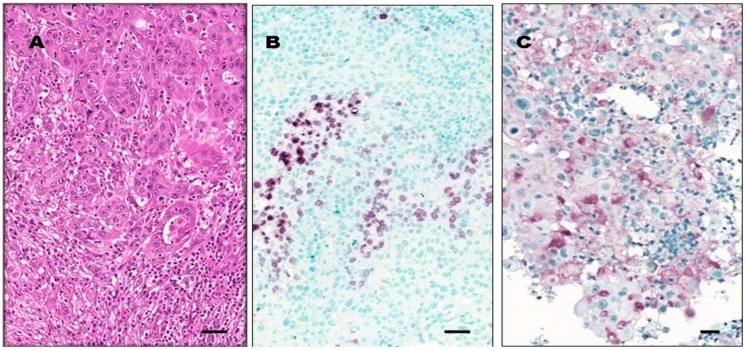
IL-6 protein expression in OSCC tumor tissue. Images from a representative tissue sample exhibiting OSCC with moderate differentiation grade (G2). (**A**) Abundant peri- and intratumoral tumor-infiltrating leukocytes (TILs) surrounding and intermixed with tumor cells (hematoxylin-eosin staining). (**B**) A region of a representative tumor sample showing many IL-6-positive TILs, whereas cancer cells are negative for IL-6 staining. (**C**) Another region of the same representative tumor sample with large squamous carcinoma cells exhibiting IL-6 positivity. Original magnification for all three images is 20×. Horizontal bars indicate 20 µm. Images (B) and (C) represent the immunohistochemistry (IHC) reaction using the peroxidase-based technique with VIP chromogen, as described in Materials and Methods.

We performed an IHC investigation of the neoplastic lesions so as to see if the IL-6 mRNA and protein detected in the saliva of patients with OSCC might be produced by cells in the tumor tissue. We detected a positive IL-6 reaction in 28/41 (68%) samples. In 16/41 samples, neoplastic cells were stained positively, and in 26/41 samples, there were detectable IL-6 protein expression in TILs (Figure 2). TILs consisted mostly of neutrophil granulocytes, CD163-positive macrophages, and CD3-positive T-lymphocytes. Occasionally, CD20-positive B-lymphocytes, plasma cells, and eosinophil granulocytes were found. CD4-positive T-cells predominated over CD8-positive T-cells. The CD4:CD8 ratio was 2:1 in 30/41 (73%) of cases. We noted a CD4:CD8 inversion (CD4:CD8 < 1:2) in 5/41 (12%) cases without signs of tumor cell destruction.

## 4. Discussion

The aim of the present investigation was to validate salivary IL-6 mRNA and protein as a diagnostic biomarker of OSCC. Saliva samples from patients with histologically proven, newly diagnosed OSCC were collected from tertiary treatment centers representing each geographic region of Hungary. The age distribution of patients was similar to previously reported Hungarian regional and nation-wide epidemiologic data. The predominance of female patients with 37% and the prevalence of advanced patients (stage 3 and 4: 62%) in the present investigation was more expressed than observed previously [1,12,20,21]. Although the distribution pattern of histological differentiation grades (G2 > G1 > G3) was similar as that reported in a previous North-Eastern Hungarian cohort, the ratio of less differentiated grades (G2 and G3) was slightly higher but the same as in the preliminary institutional pilot investigation [12,20].

Salivary IL-6 was chosen as the potentially best-performing biomarker from 9 mRNA and 14 protein molecules tested in a small-scale pilot cohort of patients from one of the participating centers (Debrecen) [12,13]. Results on quantitatively determined salivary IL-6 mRNA transcripts have not yet been published prior to our previous pilot cohort and the present multi-center study investigating the largest number of patients with OSCC. Earlier only one study reported on the expression of salivary IL-6 mRNA in patients with OSCC; however, the level of expression was not quantified [22]. We found IL-6 mRNA transcripts by qPCR in 71 of 74 saliva samples of patients with OSCC. In contrast, only 18 of 64 control saliva samples contained quantifiable levels of IL-6 mRNA transcripts, and there was a statistically significant difference in the expression levels of normalized IL-6 mRNA transcripts between patients and controls. With an AUC value of 0.9379 of the ROC curve, IL-6 mRNA is one of the best-performing salivary OSCC biomarkers reported so far [10,23,24,25]. 

At the protein level, salivary IL-6 was reported most consistently to distinguish between patients with OSCC and controls with different geographic and ethnic backgrounds. Results with other salivary protein biomarkers were found less coherent [11]. We observed significantly higher IL-6 protein concentrations in the saliva of patients with OSCC vs. controls; however, there was a partial overlap between the distribution of salivary IL-6 concentrations of patients and controls, resulting in a less favorable AUC value of the ROC curve than with salivary IL-6 mRNA. Similar salivary IL-6 concentrations to our results were observed in patients with OSCC by other investigators [4,26,27,28,29,30,31,32,33,34,35]. The attempt at normalizing salivary IL-6 protein concentration for salivary total protein concentration abolished the significant difference of salivary IL-6 manifestation observed between patients and controls (data not shown). IL-6 is a low-abundance salivary protein with a concentration in the pg/mL range, whereas salivary total protein concentration is in the mg/mL range and the low signal-to-noise ratio might obscure differences [32]. Moreover, salivary albumin, a high-abundance salivary protein, was reported to correlate with OSCC, similar to IL-6, which may further compromise normalized results [35]. In addition to OSCC, salivary IL-6 protein concentration was investigated in oral premalignant lesions (OPML). The majority of investigators found significantly increased salivary IL-6 protein concentrations in patients with OPML when compared to controls, although the concentration was less excessively elevated in OPML than in OSCC samples [26,34,36,37]. In contrast, Brailo et al. found significantly elevated IL-6 protein concentrations in salivary samples from patients with OSCC but not with OPML [31], suggesting that further studies are requested before establishing a discriminatory role of salivary IL-6 protein between OSCC and OPML. Since data on salivary IL-6 mRNA in patients with OPML are lacking, we can either confirm or refute if salivary IL-6 mRNA, although remarkably sensitive for OSCC, would be proven specific for OSCC as compared to OPML. 

Few groups investigated salivary IL-6 expression in association with various tumor stages and differentiation grades. In our study, differences both in salivary IL-6 mRNA and protein expression levels remained significant when comparing subgroups of patients with OSCC according to the stage and grade of their disease with controls, and there were no differences between distinct subgroups of patients. Similar results were reported by Sato et al. and Lisa Cheng et al. [29,32]. Three other groups observed higher salivary IL-6 concentration in patients with advanced lesions [27,32,33]. 

Conflicting results were reported on the potential influence of age, sex, smoking, and ethanol consumption habits on salivary IL-6 protein concentration in patients with OSCC [27,29,31,33,36]. Using linear regression analysis, we found significant associations between age and DMFT scores and salivary IL-6 mRNA expression confirming the results of our previous pilot studies, suggesting the influence of oral health on salivary OSCC biomarkers [12,13]. Yet, the separation between salivary mRNA levels of patients with and without OSCC is robust enough to maintain its value as an OSCC biomarker. Sex, smoking, and ethanol consumption did not bias the expression of either salivary IL-6 mRNA or IL-6 protein. 

In a search for the source of elevated salivary IL-6 levels in patients with OSCC, we investigated the expression of IL-6 protein in the tumor tissue. In the majority of cases, tumor cells, and TILs were positive for IL-6 staining. Similar observations were reported in one publication [38]. These results, together with experimental evidence, suggest that a paracrine loop of stimulation may exist between neoplastic and nonmalignant cells of the tumor microenvironment [39,40]. The positive feedback loop of stimulation may explain the significant upregulation of salivary IL6 expression in patients with OSCC both at the mRNA and protein level when compared to controls. Thus, the IL-6-IL-6 receptor interaction may offer therapeutic targets in OSCC [41]. 

In conclusion, salivary IL-6 mRNA is a robust biomarker for OSCC in the Hungarian population in comparison with tumor-free control persons. However, caution is warranted before claiming the specificity of this biomarker without investigating salivary IL-6 mRNA in patients with OPML. Further investigations, involving patients from different ethnic groups and geographic regions may prove the value of quantitative salivary IL-6 mRNA expression as a general OSCC biomarker. The relevance of salivary IL-6 mRNA in OSCC was supported by the significantly elevated salivary IL-6 protein concentration in patients with OSCC vs. controls. Moreover, intratumor IL-6 production both by tumor cells and TILs, detected by IHC, may serve as a likely source of salivary IL-6 mRNA and protein in patients with OSCC.

## Figures and Tables

**Table 1:** Demographic and clinicopathological characterization of patients with oral squamous cell carcinoma (OSCC) and control subjects.

	-	Patients (*N* = 95)	Controls (*N* = 80)	*P*
Sex	Male	60 (63%)	30 (37%)	0.004 *
	Female	35 (37%)	50 (63%)	
Age	(years; mean ± SD)	61.7 ± 9.8	61.7 ± 9.2	0.982 **
OSCC stage				
*N* = 70	St I	14 (20%)	–	na ***
	St II	22 (31%)	–	na ***
	St III	13 (19%)	–	na***
	St IV	21 (30%)	–	na ***
Histological grade				
*N* = 70	G1	17 (24%)	–	na ***
	G2	42 (60%)	–	na ***
	G3	11 (16%)	–	na ***
DMFT	(N; mean ± SD)	64; 27.6 ± 6.4	47; 24.8 ± 6.9	0.011 **
GI	(N; mean ± SD)	36; 0.53 ± 0.49	41; 0.42 ± 0.34	0.79 **
Ethanol consumption	at least once a week	46 (48%)	14 (30%)	0.035 *
	less than once a week	49 (52%)	33 (70%)	
Smoking	regular smoking	62 (65%)	8 (17%)	<0.001 *
	non-smoker or occasional smoker	33 (35%)	40 (83%)	

N = number of patients * chi-square test; ** t-test; *** not applicable.

**Table 2:** Differential expression of IL6 mRNA and IL6 protein in the saliva of patients with OSCC and controls.

	IL-6 mRNA Normalized Values				IL-6 Protein [pg/mL]			
Sample	*N*	mean	SE	*p* *	*N*	mean	SE	*p* *
OSCC								
St I-II	31	1.634	0.400	<0.001	30	59.72	18.09	<0.001
St III-IV	23	23.694	19.850	<0.001	28	102.44	28.22	<0.001
G1	9	1.440	0.501	<0.001	16	39.08	10.92	0.005
G2	34	16.327	13.550	<0.001	35	102.56	26.24	<0.001
G3	11	2.505	1.540	<0.001	7	63.58	22.45	0.010
Total	74	3.301	0.885	<0.001	74	70.98	14.06	<0.001
Control	64	0.037	0.012		71	12.45	3.29	

* Statistical comparison (Mann-Whitney U-test) of indicated groups of patient and control samples. There were no significant differences either in IL6 mRNA or IL6 protein expression between different subgroups of patients according to tumor stages and grades.

**Table 3:** Associations between demographic variables, gingival inflammation status, ethanol consumption, and smoking habits and ln-transformed salivary levels of IL-6 mRNA and IL-6 protein according to multivariate linear regression analysis.

		In Transformed IL-6 mRNA		In Transformed IL-6 Protein	
		Adjusted linear regression coefficient	*p*-value	Adjusted linear regression coefficient	*p*-value
Sex	female/male	−0.181	0.1	−0.159	0.11
**Age**	**Year**	**0.234**	**0.037**	0.174	0.094
**DMFT**		**0.234**	**0.037**	0.174	0.094
Gingival index		0.094	0.488	0.201	0.1
Alcohol consumption					
at least once a					
week/less than		0.561	0.07	−0.1	0.375
once a week					
Smoking					
regular smoking					
non-smoker or		−0.134	0.232	0.005	0.959
Occasional					
smoker					

Significant associations are indicated in bold characters.

## Supplementary Materials

The following are available online at https://www.mdpi.com/2077-0383/8/11/1958/s1, Table S1: Demographic, clinicopathological characterization and IL-6 mRNA and IL-6 protein levels of individual patients with OSCC and controls.

## References

[1] Diz P., Meleti M., Diniz-Freitas M., Vescovi P., Warnakulasuriya S., Johnson N.W., Kerr A.R. (2017). Oral and pharyngeal cancer in Europe: Incidence, mortality and trends as presented to the Global Oral Cancer Forum. Trans. Res. Oral Oncol..

[2] Zhang H., Dziegielewski P.T., Biron V.L., Szudek J., Al-Qahatani K.H., O’Connell D.A. (2013). Survival outcomes of patients with advanced oral cavity squamous cell carcinoma treated with multimodal therapy: A multi-institutional analysis. J. Otolaryngol. Head Neck Surg..

[3] Carreras-Torras C., Gay-Escoda C. (2015). Techniques for early diagnosis of oral squamous cell carcinoma: Systematic review. Med. Oral Patol. Oral Cir. Bucal..

[4] SahebJamee M., Eslami M., AtarbashiMoghadam F., Sarafnejad A. (2008). Salivary concentration of TNFalpha, IL1 alpha, IL6, and IL8 in oral squamous cell carcinoma. Med. Oral Patol. Oral Cir. Bucal..

[5] Wu J.Y., Yi C., Chung H.R., Wang D.J., Chang W.C., Lee S.Y., Lin C.T., Yang Y.C., Yang W.C. (2010). Potential biomarkers in saliva for oral squamous cell carcinoma. Oral Oncol..

[6] Panta P., Venna V.R. (2014). Salivary RNA signatures in oral cancer detection. Anal. Cell. Pathol..

[7] Khan R.S., Khurshid Z., Akhbar S., Faraz Moin S. (2016). Advances of Salivary Proteomics in Oral Squamous Cell Carcinoma (OSCC) Detection: An Update. Proteomes.

[8] Radhika T., Jeddy N., Nithya S., Muthumeenakshi R.M. (2016). Salivary biomarkers in oral squamous cell carcinoma—An insight. J. Oral Biol. Craniofac. Res..

[9] Lohavanichbutr P., Zhang Y., Wang P., Gu H., Nagana Gowda G.A., Djukovic D., Buas M.F., Raftery D., Chen C. (2018). Salivary metabolite profiling distinguishes patients with oral cavity squamous cell carcinoma from normal controls. PLoS ONE.

[10] Zhao S.Y., Wang J., Ouyang S.B., Huang Z.K., Liao L. (2018). Salivary Circular RNAs Hsa_Circ_0001874 and Hsa_Circ_0001971 as Novel Biomarkers for the Diagnosis of Oral Squamous Cell Carcinoma. Cell. Physiol. Biochem..

[11] Sahibzada H.A., Khurshid Z., Khan R.S., Naseem M., Siddique K.M., Mali M., Zafar M.S. (2017). Salivary IL-8, IL-6 and TNF-α as potential diagnostic biomarkers for oral cancer. Diagnostics.

[12] Horváth J., Szabó A., Tar I., Dezső B., Kiss C., Márton I., Scholtz B. (2018). Oral health may affect the performance of mRNA-based saliva biomarkers for oral squamous cell cancer. Pathol. Oncol. Res..

[13] Csősz É., Lábiscsák P., Kalló G., Márkus B., Emri M., Szabó A., Tar I., Tőzsér J., Kiss C., Márton I. (2017). Proteomics investigation of OSCC-specific salivary biomarkers in a Hungarian population highlights the importance of identification of population-tailored biomarkers. PLoS ONE.

[14] World Health Organization (1997). Oral Health Surveys: Basic Methods.

[15] Lang N.P., Corbet E.F. (1995). Periodontal diagnosis in daily practice. Int. Dent. J..

[16] Pindborg J.J., Reichart P.A., Smith C.J., Van der Waal I. (1997). Histological Typing of Cancer and Precancer of the Oral Mucosa. WHO International Histological Classification of Tumours.

[17] Sobin L.H., Gospodarowitz M.K., Wittekind C. (2010). Head and Neck Tumours: Lip and oral cavity. TNM Classification of Malignant Tumours.

[18] Tsakiris I., Torocsik D., Gyongyosi A., Dozsa A., Szatmari I., Szanto A., Soos G., Nemes Z., Igali L., Marton I. (2012). Carboxypeptidase-M is regulated by lipids and CSFs in macrophages and dendritic cells and expressed selectively in tissue granulomas and foam cells. Lab. Investig..

[19] Szabó K., Papp G., Dezso B., Zeher M. (2014). The histopathology of labial salivary glands in primary Sjögren’ syndrome: Focusing on follicular helper T cells in the inflammatory infiltrates. Mediators Inflamm..

[20] Nemes J.A., Redl P., Boda R., Kiss C., Márton I.J. (2008). Oral cancer report from Northeastern Hungary. Pathol. Oncol. Res..

[21] Németh Z., Turi K., Léhner G., Veres D.S., Csurgay K. (2013). The prognostic role of age in oral cancer. A clinical study. Magy. Onkol..

[22] St John M.A., Li Y., Zhou X., Denny P., Ho C.M., Montemagno C., Shi W., Qi F., Wu B., Sinha U. (2004). Interleukin 6 and interleukin 8 as potential biomarkers for oral cavity and oropharyngeal squamous cell carcinoma. Arch. Otolaryngol. Head Neck Surg..

[23] Brinkmann O., Kastratovic D.A., Dimitrijevic M.V., Konstantinovic V.S., Jelovac D.B., Antic J., Nesic V.S., Markovic S.Z., Martinovic Z.R., Akin D. (2011). Oral squamous cell carcinoma detection by salivary biomarkers in Serbian population. Oral Oncol..

[24] Gai C., Camussi F., Broccoletti R., Gambino A., Cabras M., Molinaro L., Carossa S., Camussi G., Arduino P.G. (2018). Salivary extracellular vesicle-associated miRNAs as potential biomarkers in oral squamous cell carcinoma. BMC Cancer.

[25] Feng Y., Li Q., Chen J., Yi P., Xu X., Fan Y., Cui B., Yu Y., Li X., Du Y. (2019). Salivary protease spectrum biomarkers of oral cancer. Int. J. Oral Sci..

[26] Rhodus N.L., Ho V., Miller C.S., Myers S., Ondrey F. (2005). NF-kappaB dependent cytokine levels in saliva of patients with oral paraneoplastic lesions and oral squamous cell carcinoma. Cancer Detect. Prev..

[27] Duffy S.A., Taylor J.M., Terrell J.E., Islam M., Li Y., Wolf G.T., Teknos T.N. (2008). Interleukin-6 predicts recurrence and survival among head and neck cancer patients. Cancer.

[28] Katakura A., Kamiyama I., Takano N., Shibahara T., Muramatsu T., Ishihara K., Takagi R., Shouno T. (2007). Comparison of salivary cytokine levels in oral cancer patients and healthy subjects. Bull. Tokyo Dent. Coll..

[29] Sato J., Goto J., Murata T., Kitamori S., Yamazaki Y., Satoh A., Kitagawa Y. (2010). Changes in saliva interleukin-6 levels in patients with oral squamous cell carcinoma. Oral Surg. Oral Med. Oral Pathol. Oral Radiol. Endod..

[30] Korostoff A., Reder L., Masood R., Sinha U.K. (2011). The role of salivary cytokine biomarkers in tongue cancer invasion and mortality. Oral Oncol..

[31] Brailo V., Vucicevic-Boras V., Lukac J., Biocina-Lukenda D., Zilic-Alajbeg I., Milenovic A., Balija M. (2012). Salivary and serum interleukin 1 beta, interleukin 6 and tumor necrosis factor alpha in patients with leukoplakia and oral cancer. Med. Oral Patol. Oral Cir. Bucal.

[32] Lisa Cheng Y.S., Jordan L., Gorugantula L.M., Schneiderman E., Chen H.S., Rees T. (2014). Salivary interleukin-6 and -8 in patients with oral cancer and patients with chronic oral inflammatory diseases. J. Periodontol..

[33] Arduino P.G., Menegatti E., Cappello N., Martina E., Gardino N., Tanteri C., Cavallo F., Scully C., Broccoletti R. (2015). Possible role for interleukins as biomarkers for mortality and recurrence in oral cancer. Int. J. Biol. Markers.

[34] Panneer Selvam N., Sadaksharam J. (2015). Salivary interleukin-6 in the detection of oral cancer and precancer. Asia. Pac. J. Clin. Oncol..

[35] Rao M., Ramesh A., Adapa S., Thomas B., Shetty J. (2016). Correlation of salivary levels of interleukin-6 and albumin with oral squamous cell carcinoma. J. Health Res. Rev..

[36] Juretic M., Cerovic R., Belusic-Gobic M., Brekalo Prso I., Kqiku L., Spalj S., Pezelj-Ribaric S. (2013). Salivary levels of TNF-α and IL-6 in patients with oral premalignant and malignant lesions. Folia Biol..

[37] Dikova V.R., Principe S., Bagan J.V. (2019). Salivary inflammatory proteins in patients with oral potentially malignant disorders. J. Clin. Exp. Dent..

[38] Jinno T., Kawano S., Maruse Y., Matsubara R., Goto Y., Sakamoto T., Hashiguchi Y., Kaneko N., Tanaka H., Kitamura R. (2015). Increased expression of interleukin-6 predicts poor response to chemoradiotherapy and unfavorable prognosis in oral squamous cell carcinoma. Oncol. Rep..

[39] Yamamoto T., Kimura T., Ueta E., Tatemoto Y., Osaki T. (2003). Characteristic cytokine generation patterns in cancer cells and infiltrating lymphocytes in oral squamous cell carcinomas and the influence of chemoradiation combined with immunotherapy on these patterns. Oncology.

[40] Petruzzi M.N., Cherubini K., Salum F.G., de Figueiredo M.A. (2017). Role of tumour-associated macrophages in oral squamous cells carcinoma progression: An update on current knowledge. Diagn. Pathol..

[41] Rossi J.F., Lu Z.Y., Jourdan M., Klein B. (2015). Interleukin-6 as a therapeutic target. Clin. Cancer Res..

