# Supplementary material for: Salivary IL-6 mRNA is a Robust Biomarker in Oral Squamous Cell Carcinoma
Source: J Clin Med. 2019 Nov 13;8(11):1958. doi: 10.3390/jcm8111958 (PMC6912409; doi:10.3390/jcm8111958)
Supplement: Supplementary file 1 [file jcm-08-01958-s001.pdf]

Table S1. Demographic, clinicopathological characterization and IL-6 mRNA and IL-6 protein levels of individual patients with OSCC and controls.

Sample type	Sample code	Age	Sex (M=Male, F=Female)	DMFT	Gingival index	Smoking habits (1-Regular; 2-Occasional or No)	Alcohol consumption (1-Weekly Basis; 2-Rarely or No)	Tumor Stage	Tumor Grade	IL-6 concentration (pg/ml)	IL-6 mRNA normalized
OSCC	BOC 001	59	M	19	0	1	1	II	3	15,61	4,589931453
OSCC	BOC 002	67	F	32	NA	1	2	II	2	30,00	ND
OSCC	BOC 003	61	F	13	0	2	2	I	2	1,46	1,035796387
OSCC	BOC 004	69	M	32	0	2	1	II	2	69,92	0,038904832
OSCC	BOC 005	86	F	32	0	2	2	IV	2	3,47	ND
OSCC	BOC 006	56	M	32	NA	1	2	IV	2	13,56	ND
OSCC	BOC 007	66	M	32	NA	2	1	IV	2	190,10	ND
OSCC	BOC 009	49	M	30	0	1	1	II	1	2,18	0
OSCC	BOC 010	65	M	32	0	2	2	III	2	16,04	ND
OSCC	BOC 015	52	F	32	NA	1	2	I	1	87,97	1,678196114
OSCC	BOC 016	74	F	32	NA	2	2	I	1	24,06	ND
OSCC	BOC 017	71	F	16	0,2	2	2	IV	2	445,08	0,201773205
OSCC	BOC 031	56	M	23	0,3	1	1	I	1	7,11	0,217992732
OSCC	BOC 032	50	M	28	NA	2	2	I	1	7,69	0,173759702
OSCC	BOC 033	60	F	32	NA	1	2	IV	2	58,54	15,267389
OSCC	BOC 035	65	M	NA	NA	1	2	II	2	ND	0,462466412
OSCC	BOC 037	75	F	32	NA	2	2	II	2	29,46	0,047439508
OSCC	BOC 038	74	M	32	NA	1	1	II	2	10,29	0,472281223
OSCC	BOC 039	58	M	26	0,5	1	2	IV	3	6,52	0,390736072

OSCC	BOC 040	58	F	16	0,2	2	2	II	2	29,71	0,267205328
OSCC	DOC 007	73	M	32	1	2	2	III	1	11,54	ND
OSCC	DOC 008	69	F	32	0	2	2	IV/A	1	15,45	ND
OSCC	DOC 010	67	F	32	0	1	2	IV/B	1	3,58	ND
OSCC	DOC 012	52	M	13	0	1	1	IV/A	2	0,24	ND
OSCC	DOC 014	57	M	23	0,625	1	2	III	1	23,27	ND
OSCC	DOC 015	59	F	32	NA	1	2	I	1	1,29	ND
OSCC	DOC 020	67	M	32	NA	1	2	I	NA	12,28	ND
OSCC	DOC 022	50	F	29	0,25	1	2	II	2	10,99	0,921686481
OSCC	DOC 023	52	M	32	0	1	2	IV/A	2	18,02	0,824360318
OSCC	DOC 026	48	M	25	1	2	NA	I	2	25,16	1,525045416
OSCC	DOC 039	64	M	32	0,5	1	2	II	3	174,84	0,414339007
OSCC	DOC 042	44	M	32	1	1	1	IV/B	2	318,59	1,646114981
OSCC	DOC 045	55	M	32	NA	1	2	II	1	72,97	1,907499243
OSCC	DOC 052	54	M	32	NA	1	2	IV/B	2	446,09	5,289707917
OSCC	DOC 053	44	M	17	1	1	1	III	2	55,70	0,162215665
OSCC	DOC 054	72	M	25	1	1	1	II	1	117,08	0,214819593
OSCC	DOC 056	57	F	31	2	1	NA	II	2	ND	0,484088531
OSCC	DOC 066	49	F	32	1	1	1	I	1	9,46	ND
OSCC	DOC 090	60	M	32	NA	1	1	II	2	53,54	2,533983189
OSCC	DOC 091	49	M	27	NA	2	1	III	3	54,85	0,447662748
OSCC	DOC 104	47	M	12	NA	1	1	I	3	121,85	0,160838575
OSCC	DOC 107	62	M	NA	NA	NA	1	IV	2	65,22	0,037489506
OSCC	DOC 108	48	M	NA	NA	2	2	II	3	3,26	0,002688816
OSCC	DOC 109	62	M	NA	NA	2	2	I	1	2,32	9,606098355

OSCC	DOC 110	64	M	31	1	2	2	I	3	6,16	1,145750448
OSCC	DOC 111	69	F	19	1	1	2	II	3	ND	0,472981226
OSCC	POC 001	55	M	NA	NA	NA	NA	NA	NA	ND	0,035247113
OSCC	POC 002	58	F	NA	NA	NA	NA	NA	NA	ND	20,48556828
OSCC	POC 003	63	M	NA	NA	NA	NA	NA	NA	9,76	ND
OSCC	POC 015	63	M	NA	NA	NA	NA	NA	NA	0,98	ND
OSCC	POC 016	67	M	NA	NA	NA	NA	NA	NA	ND	0,10284714
OSCC	POC 018	47	F	NA	NA	NA	NA	NA	NA	4,26	13,06348006
OSCC	POC 019	72	F	NA	NA	NA	NA	NA	NA	ND	0,157241026
OSCC	POC 020	60	M	NA	NA	NA	NA	NA	NA	60,89	ND
OSCC	POC 021	62	F	NA	NA	NA	NA	NA	NA	7,03	9,616552776
OSCC	POC 022	46	F	NA	NA	NA	NA	NA	NA	163,05	1,043386152
OSCC	POC 023	60	M	NA	NA	NA	NA	NA	NA	22,89	1,38063539
OSCC	POC 024	64	F	NA	NA	NA	NA	NA	NA	3,52	ND
OSCC	POC 025	56	M	NA	NA	NA	NA	NA	NA	ND	0,071122078
OSCC	POC 026	48	M	NA	NA	NA	NA	NA	NA	ND	0,04423906
OSCC	POC 027	64	M	NA	NA	NA	NA	NA	NA	2,34	ND
OSCC	POC 028	53	M	NA	NA	NA	NA	NA	NA	ND	5,752214266
OSCC	POC 029	60	F	NA	NA	NA	NA	NA	NA	ND	2,040802339
OSCC	POC 030	63	F	NA	NA	NA	NA	NA	NA	299,31	55,97179686
OSCC	POC 031	47	F	NA	NA	NA	NA	NA	NA	2,23	1,020513476
OSCC	POC 032	55	M	NA	NA	NA	NA	NA	NA	0,69	0
OSCC	POC 033	52	M	NA	NA	NA	NA	NA	NA	ND	1,635293853
OSCC	POC 034	46	F	NA	NA	NA	NA	NA	NA	1,77	2,325770876
OSCC	POC 035	61	M	NA	NA	NA	NA	NA	NA	4,35	0,213603936

OSCC	POC 039	75	M	NA	NA	NA	NA	NA	NA	5,65	0
OSCC	POC 040	67	M	NA	NA	NA	NA	NA	NA	3,99	ND
OSCC	SOC 001	70	F	23	NA	2	2	II	2	ND	0,357166858
OSCC	SOC 002	58	F	16	NA	2	2	IV/B	NA	ND	0,986511992
OSCC	SOC 004	59	M	32	0,5	1	2	IV/B	2	43,33	469,6886045
OSCC	SOC 008	70	F	22	0	1	1	II	1	ND	2,188225996
OSCC	SOC 009	50	M	13	0,6	1	1	IV	NA	ND	3,532889175
OSCC	SOC 011	84	F	32	NA	2	NA	II	2	156,42	0,816215719
OSCC	SOC 012	59	M	28	0	1	1	IV/C	2	330,98	0,808905535
OSCC	SOC 013	63	F	32	NA	1	2	II	2	7,56	6,54149689
OSCC	SOC 014	82	M	32	NA	2	2	IV/B	NA	73,96	9,570170536
OSCC	SOC 015	53	M	32	1	1	2	0	2	0,59	2,159519919
OSCC	SOC 016	76	M	32	NA	2	2	III	2	ND	0,376845661
OSCC	SOC 018	68	F	32	NA	1	1	I	2	4,36	1,253756451
OSCC	SOC 019	57	M	21	0,5	1	2	IV/B	2	6,53	ND
OSCC	SOC 021	59	M	28	1	1	2	IV/B	2	ND	3,636013571
OSCC	SOC 023	70	M	32	NA	2	2	IV/A	3	ND	0,744879798
OSCC	SOC 024	72	F	32	NA	2	2	II	1	111,33	5,023246231
OSCC	SOC 028	73	M	NA	NA	1	1	IV/B	1	117,97	1,560184945
OSCC	SOC 031	56	M	17	NA	1	2	III	2	53,36	ND
OSCC	SOC 032	63	M	24	0,3	2	2	II	2	ND	3,280057567
OSCC	SOC 033	57	F	32	NA	1	2	III	2	483,62	0,688595869
OSCC	SOC 034	77	F	32	1	2	NA	I	2	525,00	10,24405065
OSCC	SOC 035	67	M	32	NA	1	1	III	3	ND	18,16667187
OSCC	SOC 036	72	M	32	NA	1	2	III	2	3,55	0,37875259

OSCC	SOC 037	41	M	NA	0	2	2	III	NA	68,84	0,969328005
CTL	BOC 012	57	F	32	NA	2	2	-	-	2,52	0,092919426
CTL	BOC 013	77	M	32	NA	1	NA	-	-	2,60	0
CTL	BOC 018	60	F	32	0,4	2	1	-	-	7,43	0
CTL	BOC 019	63	M	32	NA	1	2	-	-	2,87	0
CTL	BOC 022	62	F	25	0,4	2	2	-	-	5,78	0,000734576
CTL	BOC 025	65	F	24	0,5	2	2	-	-	61,33	0,107080639
CTL	BOC 028	64	M	19	0	2	NA	-	-	3,38	0
CTL	BOC 030	72	M	27	0,1	2	1	-	-	4,64	0,000526085
CTL	DOC 027	68	F	32	0,2	2	2	-	-	17,07	ND
CTL	DOC 028	52	M	28	1	2	NA	-	-	0,89	ND
CTL	DOC 029	50	M	26	0,4	1	NA	-	-	2,97	ND
CTL	DOC 031	67	F	19	0	2	1	-	-	1,98	ND
CTL	DOC 032	59	F	26	0	1	2	-	-	11,39	ND
CTL	DOC 033	58	F	19	0	2	NA	-	-	4,65	ND
CTL	DOC 034	56	F	32	NA	2	NA	-	-	7,42	ND
CTL	DOC 035	63	F	32	NA	2	NA	-	-	0,62	ND
CTL	DOC 036	59	F	22	0	2	2	-	-	7,42	ND
CTL	DOC 037	55	F	19	0	2	2	-	-	10,86	ND
CTL	DOC 046	25	F	15	0	2	2	-	-	21,85	ND
CTL	DOC 102	65	F	32	0	NA	NA	-	-	3,69	0,030440351
CTL	DOC 103	58	F	23	0	NA	NA	-	-	7,10	0,002084019
CTL	DOC 106	60	M	NA	NA	NA	NA	-	-	1,45	0,055754541
CTL	POC 041	26	F	NA	NA	NA	NA	-	-	3,74	0
CTL	POC 042	41	F	NA	NA	NA	NA	-	-	2,68	0

CTL	POC 043	28	F	NA	NA	NA	NA	-	-	1,19	0
CTL	POC 044	30	M	NA	NA	NA	NA	-	-	ND	0,024740299
CTL	POC 045	26	F	NA	NA	NA	NA	-	-	5,84	ND
CTL	POC 046	44	M	NA	NA	NA	NA	-	-	3,37	0
CTL	POC 048	27	F	NA	NA	NA	NA	-	-	2,19	0
CTL	POC 049	29	M	NA	NA	NA	NA	-	-	7,81	0
CTL	POC 050	27	M	NA	NA	NA	NA	-	-	4,77	0
CTL	POC 051	26	F	NA	NA	NA	NA	-	-	9,38	0
CTL	POC 052	34	M	NA	NA	NA	NA	-	-	6,15	0
CTL	POC 053	31	F	NA	NA	NA	NA	-	-	3,92	0
CTL	POC 054	26	F	NA	NA	NA	NA	-	-	7,54	0
CTL	POC 055	38	F	NA	NA	NA	NA	-	-	ND	0,009284979
CTL	POC 056	33	M	NA	NA	NA	NA	-	-	3,38	ND
CTL	POC 057	27	M	NA	NA	NA	NA	-	-	3,23	0
CTL	POC 058	32	M	NA	NA	NA	NA	-	-	3,15	0
CTL	POC 059	28	F	NA	NA	NA	NA	-	-	5,38	0
CTL	POC 060	25	F	NA	NA	NA	NA	-	-	9,92	0
CTL	POC 061	41	F	NA	NA	NA	NA	-	-	8,12	0
CTL	POC 062	53	M	NA	NA	NA	NA	-	-	14,49	0,012956055
CTL	POC 063	30	M	NA	NA	NA	NA	-	-	6,67	0
CTL	POC 064	26	M	NA	NA	NA	NA	-	-	3,84	0
CTL	POC 065	51	F	NA	NA	NA	NA	-	-	4,49	0
CTL	POC 066	32	F	NA	NA	NA	NA	-	-	5,22	0
CTL	POC 067	40	M	NA	NA	NA	NA	-	-	4,20	0,004774677
CTL	POC 068	36	M	NA	NA	NA	NA	-	-	5,29	0

CTL	POC 069	43	F	NA	NA	NA	NA	-	-	6,96	0
CTL	POC 074	30	M	NA	NA	NA	NA	-	-	ND	0
CTL	SOC 010	42	F	18	0,2	2	2	-	-	0,81	0,012913541
CTL	SOC 025	71	M	NA	NA	NA	NA	-	-	13,01	0,002528937
CTL	SOC 027	64	M	NA	NA	NA	NA	-	-	1,49	0,318595771
CTL	SOC 035	53	F	NA	NA	NA	NA	-	-	ND	0,006246308
CTL	SOC 042	39	F	5	0,3	2	2	-	-	1,09	0,072759585
CTL	SOC 043	46	F	20	0,24	2	2	-	-	1,68	0,075059999
CTL	SOC 044	77	F	30	0,6	2	2	-	-	17,81	0
CTL	SOC 045	64	M	21	0,6	2	2	-	-	5,63	0
CTL	SOC 046	47	F	20	0,3	2	2	-	-	8,83	0,196902976
CTL	SOC 047	52	F	25	0,5	2	2	-	-	ND	0
CTL	SOC 048	24	F	14	0,3	1	2	-	-	6,41	0
CTL	SOC 049	69	F	32	1	2	2	-	-	2,38	0,174548171
CTL	SOC 050	49	F	15	0,3	2	2	-	-	5,77	0,462533229
CTL	SOC 051	55	M	32	0,3	2	2	-	-	6,77	0,136136148
CTL	SOC 052	49	M	19	0,3	2	1	-	-	24,23	0,005868113
CTL	SOC 053	57	F	32	NA	1	NA	-	-	ND	0,444402945
CTL	SOC 054	75	F	28	0,6	2	2	-	-	3,92	0,001878702
CTL	SOC 055	62	M	32	0,8	1	2	-	-	18,77	0
CTL	SOC 056	69	M	31	0,6	1	1	-	-	ND	0,004364828
CTL	SOC 057	69	F	31	0,5	2	2	-	-	ND	0,046115508
CTL	SOC 058	79	F	28	0,7	2	NA	-	-	ND	0
CTL	SOC 059	58	M	32	0,3	2	2	-	-	16,54	0,031596704
CTL	SOC 060	74	F	30	0,7	2	2	-	-	9,13	0,013054456

CTL	SOC 061	74	M	31	0,6	2	NA	-	-	151,16	ND
CTL	SOC 062	56	F	28	0,5	2	2	-	-	9,28	0
CTL	SOC 063	79	F	28	0,4	2	NA	-	-	8,77	0
CTL	SOC 064	76	F	22	0,5	2	NA	-	-	54,28	0
CTL	SOC 065	68	F	27	0,55	2	2	-	-	8,62	ND
CTL	SOC 067	66	F	30	0,8	6	2	-	-	174,64	ND

CTL = control; NA = not available; ND = not determined; OSCC = patient with OSCC.
